# Individual differences and counterproductive academic behaviors in high school

**DOI:** 10.1371/journal.pone.0238892

**Published:** 2020-09-10

**Authors:** Dámaris Cuadrado, Jesús F. Salgado, Silvia Moscoso

**Affiliations:** Department of Political Science and Sociology, University of Santiago de Compostela, Santiago de Compostela, Spain; Aalborg University, DENMARK

## Abstract

Counterproductive academic behaviors (CAB) is a problem that has plagued academic institutions for centuries. However, research has mostly been focused on higher learning institutes in North America. For this reason, literature on CAB must be expanded to other geographical areas and academic levels. The present research analyses the prevalence and correlates of CAB in a sample of Spanish high school students. The results indicate that CAB is a common phenomenon, cheating and low effort behaviors being the most prevalent forms. Correlational analyses revealed that conscientiousness (ρ = -.55, *p* < .01), emotional stability (ρ = .28, *p* < .01), and agreeableness (ρ = -.26, *p* < .05) are predictors of CAB. Multiple regression analyses showed that conscientiousness is the dimension exerting the strongest impact on CAB (β = -.64, *p* < .01), followed by agreeableness, and emotional stability. These three dimensions accounted for 51% of CAB variance. Last, implications for theory and practice are described.

## Introduction

There is a much greater wealth of research on counter productivity in colleges and universities than for any other level of education. One possible explanation why there have been more studies at this level could be that there are more administrative requirements and permits needed to carry out investigations in high schools and elementary schools. These sometimes-tedious procedures affect the researcher’s ability to collect data. The flexibility in the administrative processes for undergraduate and graduate students at universities and colleges as well as an easier access to, availability of, and contact with students are a few of the advantages that researchers experience when collecting data at these institutions. Nonetheless, there is empirical evidence that indicates that CAB is a prevalent problem in education at all other levels, especially at high schools.

While most of the research on CAB has been carried out at universities and colleges in North America, results indicate that younger students are also familiar with CAB practices. Rates of its occurrence among high schoolers sometimes reach alarming levels. For instance, Vinski and Shick [1] found that 88% of a sample of American high school students admitted to having cheated on tests at least once, 42% said they have done so several times, and 16% to have done it many times. Some of the most shocking results are those reported by the last Report Card on the Ethics of American Youth [2]. After having administered a survey to a sample of more than 23,000 American high school students, results indicated that 51% of the sample admitted to having cheated on a test, 55% to having lied to a teacher about something significant, and 75% to have copied another classmate’s homework.

Prevalence of CAB in high schools has also been analyzed using retrospective questionnaires. For instance, Colmar Brunton [3] found that 52% of the adults assessed admitted to having copied in examinations or having cheated on academic projects while in high school. A similar result was found by Lai and Weeks [4]. In their study they found that 50% of 241 university students confessed to having engaged in plagiarism at least once in high school.

In Europe, research on the topic is less prominent. However, results show that CAB among high school students is also a worrying issue. For instance, the study by Farkas and Orosz [5] reports high percentages of a set of self-reported negative behaviors occurring among 236 Hungarian high school students. The results show that 67.1% of the surveyed students had used prohibited notes during examinations and that 53.4% had copied off a classmate’s exam on at least one occasion. In Russia, Poltorak [6] found that 16.1% of the 247 high schoolers studied had cheated on written exercises, 30% had done so in examinations and that 37.9% had done likewise in written academic projects. The results by Rujoiu [7] are especially dramatic. Out of a sample of 254 Romanian high schoolers, analyses indicated that 76.8% had helped a colleague to pass an exam by using fraudulent means, that 83.5% had cheated in examinations, and that almost the entire sample (95.2%) had taken information from the internet without mentioning the source.

Research on CAB among Spanish high school students is especially limited. One of the few studies analyzing this phenomenon is that of Sureda, Comas, and Oliver [8] on plagiarism. Their findings revealed that 47.4% of the sample, on average, has committed at least once acts of plagiarism. Another study of note is the one by Clariana, Gotzens, Badia, and Cladella [9] which examined the relationship between plagiarism and procrastination. Mut, Morey, and Vázquez [10] also expanded the research on this topic by examining the past experiences of teachers in secondary education on detecting plagiarism.

In conclusion, evidence shows that CAB prevalence reaches concerning rates at the high school level in many countries other than the US and Canada with different educational systems and cultures. This situation is actually aggravated since the consequences of CAB jeopardize the reputation of the educational system, negatively affect a student's academic performance, undermine the morale of teachers and other students, and might lead to similar counterproductive behaviors in organizational contexts after graduation [11–15]. Consequently, to address the occurrence of academic counterproductivity and to empirically study its potential correlates is of the upmost importance.

For the above reasons and to contribute to the knowledge on CAB in high school in countries others than the US and Canada, the present research has three main goals: (1) to determine to what extent overall CAB and its facets occur among a Spanish sample of high school students; (2) to find out the true relationship between the Big Five personality dimensions, GMA, and CAB; and (3) to carry out a multiple regression analysis that shows the relative weight of each predictor and the proportion of the CAB variance that is explained.

### Correlates of CAB: Big Five and GMA

Among the correlates of CAB, many variables have been tested with different predictive effectiveness. Basically, these variables have been classified into two categories: contextual characteristics (e.g., classroom size, school size, public vs. private schools, academic workload, teaching style) and individual differences (e.g., personality variables, cognitive variables, demographic characteristics).

The current research focuses on the second group of characteristics, especially in the study of the Big Five personality dimensions and the students’ general mental ability (GMA) and their relationship with CAB and its facets. The association between these variables has been previously tested in various meta-analyses [see, 16–19]. However, these meta-analyses reveal the dearth of investigation of CAB at the high school level. Credé et al. [16] analyzed the relationships between academic absenteeism and a wide range of variables such as the Big Five dimensions and intelligence. However, they only investigate samples from colleges and universities. Giluk and Postlethwaite [18] focused on the relationships between the Big Five dimensions and a compound of negative behaviors, mostly cheating and plagiarism. Out of the 18 effect sizes accumulated, only 2 were calculated using high school samples. Moreover, emotional stability was the only personality dimension analyzed. The meta-analysis by Paulhus and Dubois [19] examined the association between cheating and intelligence. They found 22 effect sizes of this relationship. However, none of them was calculated using high school samples, but only students from higher or lower academic levels. Cuadrado et al. [17] examined the link between the Big Five model dimensions, intelligence, and overall CAB and its facets. This work improved upon previous meta-analysis by integrating a larger number of studies, applying new artifactual corrections, and considering the educational level as a possible moderating variable. Their findings showed that the amount of studies carried out at the high school level was up to almost 9 times less than those found for the higher education level. Furthermore, most of the high school samples integrated fit into the North American context. The percentages of samples from the US and Canada ranged from 43% for the emotional stability-CAB relationship to 70% for the association between conscientiousness and CAB. These findings suggest once more that research must be expanded to samples of high school students from different countries.

#### Evidence on the Big Five-CAB relationship

Empirical evidence on the relationship between the Big Five model of personality and CAB shows that conscientiousness and agreeableness are the personality dimensions most linked to these behaviors. Cuadrado et al. [17] found that both dimensions were valid predictors of overall CAB with true effect sizes of -.28 and -.14, respectively. When the moderator variable “academic level” was analyzed, results for high school samples were very similar, reaching a true effect size of -.24 for conscientiousness and of -.13 for agreeableness. The results for the remaining dimensions were close to zero in both the overall analyses and in the analyses with high school samples. However, extraversion appeared as a predictor of cheating behaviors when the different factors of CAB were separately analyzed, with a true effect size of .19.

Prior research on the topic had reported similar results. For instance, Giluk and Postlethwaite [18] found conscientiousness and agreeableness to be the strongest predictors of academic dishonesty. Credé et al. [16] found that conscientiousness stood out among the remaining four personality dimensions in the prediction of academic absenteeism.

To sum up, meta-analytical results indicate that those students scoring high in conscientiousness and agreeableness are less prone to behave in a counterproductive manner in the academic context. Theoretically, conscientious people are organized, self-disciplined, planned, task-oriented, responsible, and dutiful [20–25]. Meanwhile, agreeable people are characterized as warm, altruistic, empathic, and gentle, and are additionally known for being reliable, trustful, straightforward, compliant with rules, and inoffensive to others [20–25]. Given the nature of these personality dimensions, it is not surprising that those students higher in conscientiousness and agreeableness appear to be less predisposed to engage in CAB. Regarding extraversion, empirical evidence indicates that extraverted students cheat on exams more than their introverted peers. A theoretical explanation could be the tendency of extraverts to be gregarious and sociable [20–25], traits which might manifest in a preference for more social, interactive, and exciting activities than those of an academic nature (e.g., studying for tests). Furthermore, they are often described as daring, reckless, and risk-seekers, characteristics which would make them less hesitant to play fast and loose with the rules during examinations. Additionally, Matthews, Davies, Westerman, and Stamers [26] state that extraverts often perform worse than introverts in long, monotonous, and tedious tasks (e.g. academic tasks). Hence, they might need to rely on fraudulent means such as cheating to successfully pass their examinations.

Based on the empirical evidence and the theoretical rationale presented above, our hypotheses are:

*Hypothesis 1*. Conscientiousness negatively correlates to CAB and its facets.

*Hypothesis 2*. Agreeableness negatively correlates to CAB and its facets.

*Hypothesis 3*. Extraversion positively correlates to cheating behaviors.

#### Evidence on the GMA-CAB relationship

It was suggested that individuals high in cognitive ability are more likely to anticipate, assess, and understand the consequences and negative side-effects of their actions [27]. The fact that an individual is aware of the negative consequences (e.g., being suspended, repeating a test, failing a course) of his or her actions (e.g., cheating in examinations, plagiarizing a work, lying to a teacher) might encourage him or her to experience a feeling of aversion towards behaving in a deviant manner. In the academic field, this would translate to a higher rejection of CAB involvement. Furthermore, since intelligence has proven to be an excellent predictor of academic success, intelligent students might be those who need not engage in fraudulent means to achieve good academic results [28–31].

Meta-analytical evidence supports a negative relationship between these variables. Paulhus and Dubois [19] have reported an observed negative effect size between intelligence and a compound of dishonest conducts of *r* = -.21. Meta-analysis by Cuadrado et al. [17] found that intelligence was a valid predictor of overall CAB with true validity coefficient of -.19. When samples composed of high school students were separately analyzed, the result was slightly higher in magnitude (ρ = -.22).

Cuadrado et al. [17] also performed separate analyses to test the possible differences in the results when the type of intelligence test and the intelligence construct assessed were treated as potential moderator variables. The results showed that traditional intelligence tests (e.g., Raven’s Matrices Test, Cattell’s Fair Culture Tests, Wonderlic Personnel Test) yielded a stronger result than cognitive tests used for academic admissions (e.g., SAT, ACT, GRE) (ρ = -.26 and ρ = -.13, respectively). In a similar vein, Credé et al. (2010) found that intelligence, when measured using traditional tests, negatively correlated with academic absenteeism (ρ = -.11). In contrast, the result was almost null for the cognitive tests used for academic admissions. Cuadrado et al. [17] also found that GMA tests were the ones producing the highest results in comparison with fluid or crystallized intelligence tests. The results showed that GMA tests were valid predictors of CAB (ρ = -.31). Results for crystallized intelligence measures were lower in magnitude (ρ = -.19) and for the fluid measures were almost null (ρ = .04). As the authors point out, while intelligence measures used for academic admissions, measures of fluid intelligence, and measures of crystallized intelligence evaluate a narrower range of abilities, GMA measures are broader in scope and might include the abilities necessary to avoid and inhibit the need or desire to engage in deviancy. For these reasons, out last hypothesis is:

*Hypothesis 4*. Intelligence, measured with a GMA test, negatively correlates to CAB and its facets.

## Method

### Participants and procedure

The principals and principals’ assistants of various Spanish high schools were contacted to carried out this research. Permission was requested to use the different measures and evaluate the variables of interest. Out of the fifteen high schools contacted, seven agreed to participate and provided consent to carry out the study. The goal of the research was explained to both the administrators and the students. In order to make students feel more motivated to admit their participation in counterproductive academic behaviors, we presented some of the main empirical findings on the topic showing that almost every student engages in such behaviors at some point, whether it is in high school or at other educational levels. We also clarified our interest in linking some individual characteristics to the phenomenon and made clear that all the information would be confidential and that in no case it would be treated at the individual level, but at the aggregate level.

Tests were agreed to be completed during class time. Initially, three measures were designed to be used: a personality test, an intelligence test, and a scale of academic counterproductivity. However, due to time restrictions in the high schools, in some cases, the students’ availability corresponded to just one class session, that is, fifty minutes. In those cases, only two questionnaires could be completed: the intelligence test and the CAB scale. The final sample was composed of 240 students, 106 of which were women. The average age was 17.4 years old (*SD* = 0.90), ranging from 16 to 20. The students were enrolled in the last two years of high school. 19% were in their 1^st^ year of Baccalaureate, corresponding to the eleventh grade in the American educational system (junior students), and 81% were in their 2^nd^ year of Baccalaureate (twelfth grade or senior students). The sample size for the personality measure was *N* = 126 (62 women) with an average age of 17.2 years old (*SD* = 0.96). In order to check if there would be substantial differences between the entire sample and the restricted sample of the personality measure, we performed descriptive statistics for every variable with both sets of subjects. The results indicated that the minimum and maximum values as well as the means and standard deviations were very similar in every case.

All the subjects provided their written and informed consent to participate in the study. Given the fact that the data provided by the students were confidential, that their treatment was anonymous and at the variable level, and that they were exclusively used for the current study, high school administrators declared this research exempt from parents’ approval. Moreover, the Bioethics Committee of the University of Santiago de Compostela declared this research free from the need of approval for the same reasons cited above.

### Materials

#### The Big Five model of personalit

The test IP/5F developed by Salgado [32] was used to assess the student’s personality. This inventory evaluates the five dimensions of the Big Five model (emotional stability, extraversion, openness to experience, agreeableness, and conscientiousness) using 200 items (40 items per dimension) that are grouped in 29 homogeneous-item clusters. Students had to indicate their degree of agreement with each statement by using a three-points scale (1 = *in disagreement*, 2 = *indecisive*, 3 = *in agreement*). Some examples of items are: “I hardly ever get nervous” (emotional stability), “I am not ashamed to speak in public” (extraversion), “I am a very imaginative person” (openness to experience), “I think that, in general, people are honest” (agreeableness), and “I am a meticulous person” (conscientiousness).

The IP/5F test has shown optimal psychometric properties. The internal consistency for emotional stability, extraversion, openness to experience, agreeableness, and conscientiousnes was .90, .86, .80, 74, and .87 for the normative sample (*N* = 760). Temporal stability coefficients for one year were .91, .90, .79, .65, and .72 for the same factors. The reliability data for the clusters ranged from .57 for tolerance (agreeableness) to .84 for anxiety (emotional stability). The test has also shown evidence of convergent and discriminant validity with other personality measures that evaluate the same dimensions, such as the HPI [33, 34], the NEO-FFI and the NEO-PI-R [20], the EPI [35], and the 16PF [36]. For example, the correlations between the IP/5F and the NEO-FFI were found to be .70, .88, .55, .55, and .58 for emotional stability, extraversion, openness to experience, agreeableness, and conscientiousness, respectively. Salgado, Moscoso, and Lado [37] also found that the correlations between the HPI and IP/5F were .75 for emotional stability, .69 and .74 for extraversion (HPI divides this dimension into two subfactors), .85 for openness to experience, .51 for agreeableness, and .67 for conscientiousness.

In the current study, the Cronbach alpha coefficients were very similar to those obtained for the normative sample. They were .89, .88, .85, .71, and .84 for emotional stability, extraversion, openness to experience, agreeableness, and conscientiousness.

#### GMA (general mental ability)

A Spanish adaptation of the Wonderlic Personnel Test [38] was used to assess GMA. This measure consists of 50 items that evaluate the subjects in a variety of abilities by using disordered phrases, numerical comparisons, numerical series, geometric figures, nominal parallelisms, and other problems that require a logical solution. Students had 12 minutes to correctly solve the greatest number of problems.

The Wonderlic Personnel Test is one of the most used instruments to assess general mental ability for practical and theoretical purposes. The manual of the original version shows evidence of its predictive validity regarding multiple criteria, both in the occupational and in the educational field. For instance, the correlations with training success, occupational performance, and academic performance were .67, .63, and .52, respectively. The Cronbach alpha coefficients for the normative data range from .77 to .89 depending on the test version. In the current sample, the Cronbach alpha coefficient was .70.

#### CAB (counterproductive academic behaviors)

CAB was measured using the CDAN scale [39, Negative Academic Performance Questionnaire]. This instrument consists of 30 items structured in five dimensions that assess a wide range of counterproductive behaviors in the academic context. The dimensions are cheating, misuse of resources, absenteeism, breach of rules, and low effort. Examples of items for each dimension are: (1) “I have peeked at a classmate’s exam to get the answer” (cheating); (2) “I have stolen something (e.g., a book, a notebook)” (misuse of resources); (3) “I have left the class without giving a fair reason before it ended” (absenteeism); (4) “I have used other people's work as my own” (breach of rules); and (5) “I have gone to class or to an examination poorly prepared without a fair reason” (low effort). Students had to indicate the frequency with which they have engaged in the 30 described behaviors using a five-points scale (1 = *never* to 5 = *always*). The Cronbach alpha coefficient for the overall scale was .92. For the dimensions of cheating, misuse of resources, absenteeism, breach of rules, and low effort, the coefficients were .80, .78, .81, .69, and .84.

## Results

### Descriptive statistics of CAB

The first goal of the study was to estimate the levels of prevalence of counterproductive academic behaviors among the high school students composing the sample. The descriptive statistics of the CAB variable appear in Table 1. The rows of the table represent, for the overall CAB scale and each of the five factors, the mean score, the standard deviation, the maximum score, the minimum score, and the percentage of students that have never, hardly ever, sometimes, usually, and always engaged in the different CAB factors.

**Table 1:** CAB descriptive statistics (*N* = 238).

	Overall CAB	Cheating	Misuse of resources	Absenteeism	Breach of rules	Low effort
**Mean**	64.69	15.46	8.67	13.25	11.55	15.76
***SD***	15.33	3.95	2.87	4.42	3.75	4.64
**Max.**	113.00	27.00	20.00	28.00	23.00	29.00
**Min.**	35.00	6.00	6.00	6.00	6.00	6.00
**Frequency (%)**						
Never	-	17.37	67.86	31.58	45.80	18.63
Hardly ever	-	27.03	21.85	31.01	26.68	26.75
Sometimes	-	38.52	8.54	25.98	19.19	33.89
Usually	-	13.52	1.40	7.77	5.88	14.78
Always	-	3.57	0.35	3.64	2.45	5.95

Ranging from 30 (every conduct is marked with a 1 = *never*) to 150 (every conduct is marked with a 5 = *always*), the mean score for overall CAB was 64.69 and the standard deviation 15.33. For the specific dimensions of the scale, ranging from 6 to 30, results indicate that the most prevalent form of CAB is low effort, with an average score of 15.76 (*SD* = 4.64), closely followed by cheating (*M* = 15.46, *SD* = 3.95). The results also show that the least prevalent form of CAB is the inappropriate use of resources. In this case, the mean value was 8.67 and the standard deviation was the lowest of the scale (*SD* = 2.87), meaning this is the CAB factor in which students’ scores where the most similar.

With regards to the percentage of students engaging at each level of frequency in each CAB factor, the results show that low effort behaviors are the most repeatedly performed conduct among the students of the current sample, with 87.37% admitting to having shown low effort tendencies at some point during their studies. Out of this percentage, 20.73% behave in this manner usually or always. Results for the cheating factor were very similar. 72.64% of the sample reports on having cheated at least once. Of those, 38.52% cheat sometimes and 17.09% do it often or always. Absenteeism and breach of rules also show similar rates; 68.41% and 54.20% of the sample admit to having engaged in these behaviors in high school. Out of those, 11.41% and 8.33% engage in absenteeism and break the rules often or always. The inappropriate use of resources was the least frequent CAB facet among the students sampled. 67.86% of them have never misused academic resources in high school.

Overall, descriptive analyses inform of a troubling situation in which a high percentage of students have engaged in a wide variety of counterproductive behaviors during high school.

### Correlational results

Table 2 reports on the observed Pearson correlations among the variables. The first two columns show the mean and standard deviation for every variable. Next, correlations for sex, age, the Big Five dimensions of personality, GMA, overall CAB, and its facets are presented. In the diagonal, the Cronbach alpha coefficients are displayed for each variable.

**Table 2:** Observed correlations among the variables.

	*Mean*	*SD*	Sex	Age	ES	EX	OP	A	C	GMA	CAB	CH	MR	AB	BR	LE
**1. Sex**	*0*.*56*	*0*.*50*	-													
**2. Age**	*17*.*41*	*0*.*90*	-.08	-												
**3. ES**	*32*.*09*	*14*.*84*	-.44**	-.01	.*89*											
**4. EX**	*48*.*93*	*12*.*40*	-.01	-.01	.29**	.*88*										
**5. OP**	*50*.*61*	*11*.*49*	.17	.04	.06	.48**	.*85*									
**6. A**	*39*.*76*	*8*.*43*	.03	-.10	.05	.06	.17	.*71*								
**7. C**	*31*.*87*	*11*.*41*	.18*	-.14	-.20*	-.10	.06	-.22*	.*84*							
**8. GMA**	*20*.*24*	*4*.*36*	-.27**	.06	.30**	.25**	.22*	-.02	-.18	.*70*						
**9. CAB**	*64*.*69*	*15*.*33*	-.35**	.20**	.20*	.13	-.06	-.15	-.34**	.06	.*92*					
**10. CH**	*15*.*46*	*3*.*95*	-.12	.21**	.14	.27**	-.02	-.03	-.21*	-.02	.73**	.*80*				
**11. MR**	*8*.*67*	*2*.*87*	-.34**	.01	.17	.09	-.06	-.19*	-.16	.08	.73**	.43**	.*78*			
**12. AB**	*13*.*25*	*4*.*42*	-.27**	.22**	.15	.09	.01	-.10	-.37**	.05	.84**	.49**	.54**	.*81*		
**13. BR**	*11*.*55*	*3*.*75*	-.28**	.20**	.09	.04	-.12	-.28**	-.12	.05	.82**	.54**	.61**	.63**	.*69*	
**14. LE**	*15*.*76*	*4*.*64*	-.36**	.11	.22*	.04	-.07	-.05	-.44**	.09	.76**	.40**	.40**	.58**	.47**	.*84*

*N* Big Five Factors—CAB = 124; *N* GMA—CAB = 232; *N* among the Big Five = 126; *N* among overall CAB and its dimensions: = 238; *N* GMA = 234; Cronbach alfa coefficients are presented in the diagonal; *M* = Mean score of the variable; *SD* = standard deviation of variables’ scores; Sex was coded 0 for male and 1 for female; ES = emotional stability; EX = extraversion; OP = openness to experience; A = agreeableness; C = conscientiousness; GMA = general mental ability; CAB = counterproductive academic behaviors; CH = cheating; MR = misuse of resources; AB = absenteeism; BR = breach of rules; LE = low effort.

*p < .05.

**p < .01.

As can be seen, there is a negative and significant correlation between sex and overall CAB (*r* = -.35, *p* < .01), showing that men are more prone to behave in a negative manner when compared to women. With regard to the CAB factors, there was also a negative relationship in all the cases. Despite the fact that the cheating dimension did not show a significant result (*r* = -.12, *p* > .05), the remaining facets correlated inversely and significantly with sex, ranging from *r* = -.36 (*p* < .01) for low effort to *r* = -.27 (*p* < .01) for absenteeism. These results are larger in size than those found in the meta-analysis by Whitley, Nelson, and Jones [40], who reported an observed effect size of *r* = -.08 (*K* = 34), meaning that men were also the most likely to commit CAB.

For the variable age, our results indicate that older students are more prone to engage in CAB. The observed correlation was *r* = .20 (*p* < .01). This result has a similar magnitude to that found in the meta-analysis by Whitley [41] of *r* = -.27 (*K* = 11, *N* = 3,204), where older students showed to be also more inclined to participate in academic counterproductivity.

Since the results presented in the table are observed validity coefficients, we have proceeded to correct the correlations of CAB with the Big Five personality dimensions, and GMA for artifactual errors. It is known that uncorrected correlations are affected by artifacts that exert a negative impact in their magnitude [50]. Therefore, Table 3 shows the results after these errors have been controlled.

**Table 3:** Corrected correlations of CAB and its facets with the Big Five and GMA.

	*r*_cy_	*r*_op_		95% CI (*r*_op_)				ρ		95% CI (ρ)		
				Lower		Upper				Lower	Upper	
**Emotional Stability**												
**Overall CAB**	.21*	.27*	.058		.475		.28**		.075			.488
Cheating	.16	.20	-.015		.418		.21		-.003			.428
Misuse of resources	.19	.25*	.034		.457		.26**		.051			.470
Absenteeism	.17	.21	-.001		.429		.23*		.013			.441
Breach of rules	.11	.14	-.083		.362		.15		-.074			.370
Low effort	.24*	.31**	.102		.507		.32**		.122			.522
**Extraversion**												
**Overall CAB**	.14	.19	-.047		.429		.20		-.035			.439
Cheating	.30**	.41**	.211		.609		.43**		.242			.627
Misuse of resources	.10	.14	-.099		.387		.15		-.089			.396
Absenteeism	.10	.14	-.102		.385		.15		-.092			.393
Breach of rules	.05	.07	-.180		.316		.07		-.176			.320
Low effort	.04	.06	-.186		.311		.07		-.182			.315
**Openness to Experience**												
**Overall CAB**	-.06	-.08	-.314		.148		-.09		-.320			.141
Cheating	-.02	-.03	-.262		.204		-.03		-.264			.201
Misuse of resources	-.07	-.09	-.320		.141		-.10		-.328			.133
Absenteeism	.01	.02	-.218		.247		.02		-.127			.249
Breach of rules	-.14	-.19	-.412		.034		-.21		-.427			.015
Low effort	-.08	-.10	-.330		.130		-.11		-.339			.120
**Agreeableness**												
**Overall CAB**	-.16	-.22	-.455		.016		-.26*		-.031			-.490
Cheating	-.03	-.05	-.298		.201		-.06		-.306			.193
Misuse of resources	-.22*	-.30**	-.076		-.522		-.35**		-.139			-.564
Absenteeism	-.11	-.16	-.400		.086		-.19		-.426			.054
Breach of rules	-.34**	-.45**	-.266		-.642		-.53**		-.360			-.695
Low effort	-.06	-.08	-.327		.170		-.09		-.340			.156
**Conscientiousness**												
**Overall CAB**	-.35**	-.51**	-.321		-.695		-.55**		-.373			-.722
Cheating	-.24*	-.35**	-.121		-.584		-.38**		-.157			-.606
Misuse of resources	-.18	-.28*	-.028		-.523		-.30**		-.057			-.543
Absenteeism	-.41**	-.58**	-.410		-.740		-.62**		-.467			-.766
Breach of rules	-.14	-.22	-.478		.036		-.24*		-.012			-.496
Low effort	-.48**	-.65**	-.511		-.787		-.69**		-.571			-.813
**General Mental Ability**												
**Overall CAB**	.06	.09	-.091		.266		.11		-.073			.282
Cheating	-.02	-.03	-.211		.149		-.04		-.218			.142
Misuse of resources	.09	.13	-.050		.303		.15		-.025			.325
Absenteeism	.06	.08	-.100		.257		.09		-.086			.270
Breach of rules	.06	.08	-.095		.262		.10		-.077			.278
Low effort	.10	.14	-.039		.312		.16		-.012			.337

*N* = 124 for the CAB-personality relationships; *N* = 232 for the CAB-GMA relationships; *r*_cy_ = effect size corrected for measuremnet error in the criterion; *r*_op_ = operational validity; 95% CI (*r*_op_) = lower and upper limits of the 95% confidence interval of the operational validity; ρ = true effect size; 95% CI (ρ) = lower and upper limits of the 95% confidence interval of the true validity.

*p < .05.

**p < .01.

The first column presents the corrected correlations for measurement error in the criterion (*r*_cy_), followed by the operational validity (*r*_op_), that is, the validity coefficient corrected for measurement error in the criterion and indirect range restriction in the predictor, and the lower and upper limits of the 95% confidence interval of the operational validity (CI 95% *r*_op_). Next, the true validity (ρ) is presented, that is, the validity coefficient corrected for measurement error in the predictor and criterion variables and indirect range restriction in the predictor. The two last columns are the lower and upper limits of the 95% confidence interval of the true validity (CI 95% ρ). The operational validity and true validity coefficients must be considered for different purposes. For applied purposes, operational validity is the appropriate coefficient to focus on because, in real testing situations, observed test scores are used to predict future performance (e.g., engagement in counterproductive academic behaviors) [42]. This is the reason why operational validity is the validity coefficient corrected for measurement error in the criterion but not in the predictor. However, as Schmidt and Hunter [42] point out, it is the construct-level correlation which is needed for theory testing. Hence, the true validity (ρ) is reported. The true validity coefficient is needed because it controls the underestimation produced by measurement error in X and Y and indirect range restriction in X. Furthermore, as it will be explained in the next section, ρ is the appropriate coefficient to use when multiple regression analyses are carried out [42–44].

As noted by Thorndike [45], in validity studies in employment and educational contexts, range restriction is indirect in most cases. To apply corrections for indirect range restriction, we first corrected the measurement error. Next, using the procedure of Schmidt and Hunter [42], range restriction was corrected using the *U* coefficients that were calculated using the standard deviations of the non-restricted population (the normative sample of the Big Five and GMA measures) and the standard deviations obtained in the sample of the current study. Our comments will focus on the true correlation (ρ).

#### Big Five and CAB

Starting with emotional stability, the results show a positive and significant relationship with overall CAB. The true validity was ρ = .28 (*p* < .01). The 95% confidence interval did not include zero, meaning that emotional stability is a valid predictor of CAB. For the different CAB factors, the results were positive in all the cases, ranging from ρ = .15 (*p* > .05) for breach of rules to ρ = .32 (*p* < .01) for low effort. Additionally, emotional stability appeared to be a valid predictor of three out of the five CAB dimensions. These were low effort, absenteeism, and misuse of resources (ρ = .32, *p* < .01, ρ = .23, *p* < .05, and ρ = .26, *p* < .01, respectively).

Extraversion emerged as a direct correlate of overall CAB, with an effect size of ρ = .20. However, the 95% confidence interval included zero. In regard to the specific CAB factors, extraversion showed a strong and positive correlation with cheating behaviors, reaching an effect size of ρ = .43 (*p* < .01), and being the only case in which extraversion appeared as a valid predictor. This finding supports Hypothesis 3. The remaining correlations were still positive but had a lower magnitude, ranging from ρ = .07 (*p* > .05) for breach of rules and low effort to ρ = .15 (*p* > .05) for absenteeism and misuse of resources.

Openness to experience yielded the lowest correlations. Neither in regard to the overall CAB measure, nor for its facets does it appeared as a valid predictor.

Agreeableness showed a negative relationship with both overall CAB and its facets. These findings support Hypothesis 2. For the general CAB measure, the result was a significant correlation of ρ = -.26 (*p* < .05). Agreeableness also appeared to be a valid predictor of misuse of resources (ρ = -.35, *p* < .01) and of breach of rules (ρ = -.53, *p* < .01). For the remaining CAB facets, coefficients were negative but not significant and ranged from ρ = -.19 (*p* > .05) for absenteeism to ρ = -.06 (*p* > .05) for cheating.

Last, conscientiousness appeared as the strongest predictor of CAB. The true validities were all negative and significant in all the cases. For the overall CAB measure the result was ρ = -.55 (*p* < .01), and for the remaining dimensions, the results ranged from ρ = -.24 (*p* < .05) for misuse of resources to ρ = -.69 (*p* < .01) for low effort behaviors. These results support Hypothesis 1.

In summary, the Big Five model appeared to be related to CAB behaviors. Particularly, conscientiousness, emotional stability, and agreeableness were the strongest predictors of CAB. Extraversion also appeared to be a good predictor of cheating conduct.

#### General mental ability and CAB

Contrary to our expectations, GMA did not appear as a valid predictor neither of the overall CAB measure, nor of its facets. The true correlation between GMA and overall CAB was ρ = .11 (*p* > .05), ranging for the different facets between ρ = -.04 (*p* > .05) for cheating behaviors and ρ = .16 (*p* > .05) for low effort. Hence, Hypothesis 4 is not supported.

### Linear multiple regression analyses

Aiming to test the directionality of the correlational results and the predictive weight of the variables most strongly related to CAB, we carried out linear multiple regression analyses. Based on the results presented in the previous section, our model tests the relative predictive weight and the multiple validity of conscientiousness, emotional stability, and agreeableness on overall CAB. Extraversion and openness to experience were not included in the tested model since the correlations obtained in both cases were low or very low and non-significant. In order to carry out the analyses, a matrix of corrected correlations among the variables was considered as the input data (see Table 4).

**Table 4:** Entrance matrix of true correlations.

	C	ES	A	CAB
**C**	-			
**ES**	-.23*	-		
**A**	-.28*	.06	-	
**CAB**	-.55**	.28**	-.26*	-

*N* = 124; C = conscientiousness; ES = emotional stability; A = agreeableness; CAB = counterproductive academic behaviors.

*p < .05.

**p < .01.

**Fig 1:**
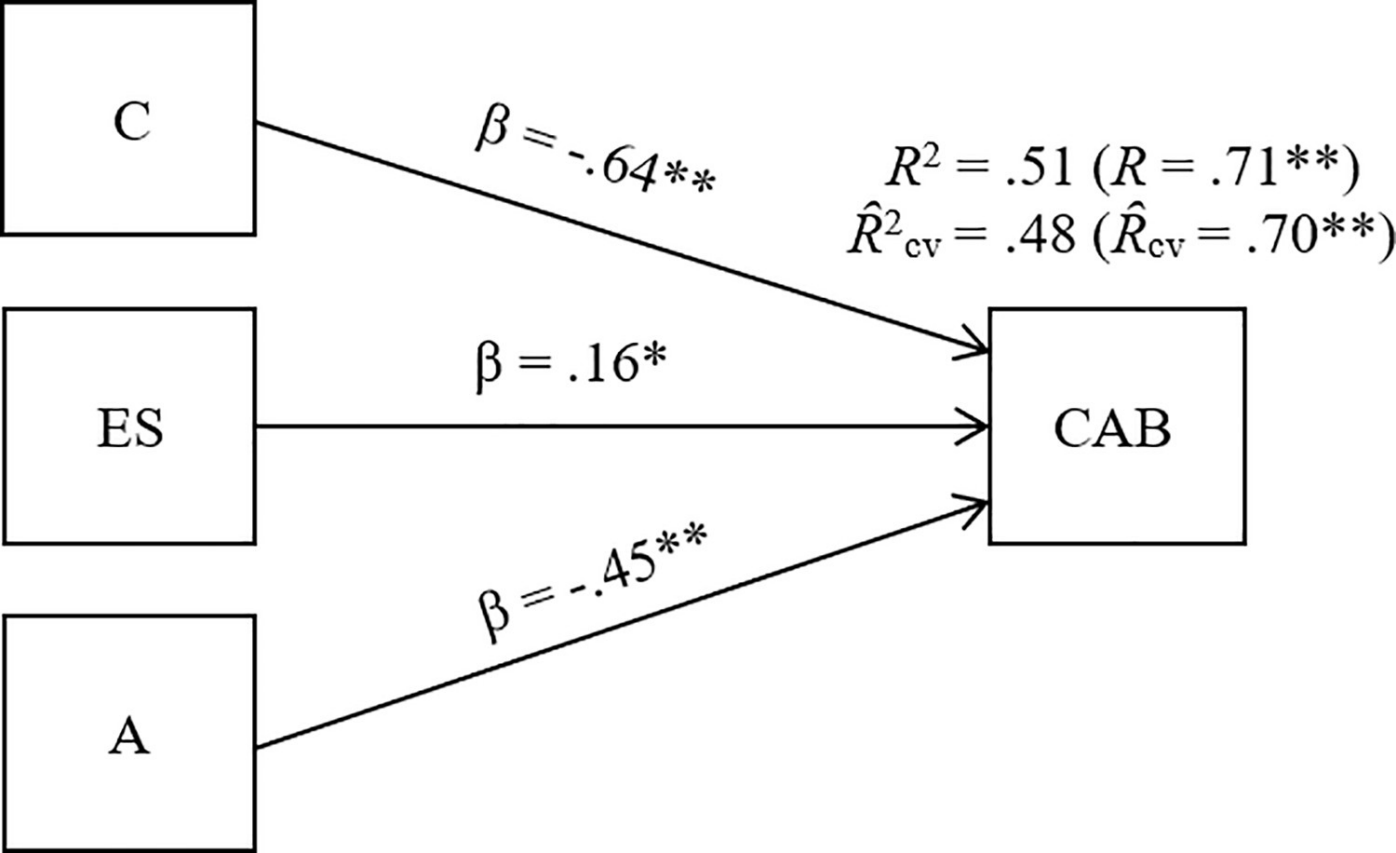
Graphical representation of the model. C = conscientiousness; ES = emotional stability; A = agreeableness; CAB = counterproductive academic behaviors. *p < .05. ** *p* < .01.

As pointed out earlier, it is known that multiple regression and structural equation modeling are meant to be applied with corrected data because the presence of artifactual errors such as measurement error and range restriction violate the independence-of-errors assumption and, consequently, the parameters could be biased [42–44]. The software LISREL 8.2 [46] was used to estimate the regressions. The obtained results are displayed in Table 5 and graphically presented in Fig 1.

**Table 5:** Regression analyses of conscientiousness, emotional stability, and agreeableness on CAB.

Variables	β	*R*	*R*^*2*^	*Ȓ*_*cv*_	*Ȓ*^*2*^_*cv*_
**Conscientiousness**	-.64**				
**Emotional Stability**	.16*				
**Agreeableness**	-.45**				
**C + ES+ A**		.71**	.51	.70**	.48

β = standardized regression coefficient*; R =* multiple regression coefficient*; R*^*2*^ *=* squared multiple regression coefficient*; Ȓ*_*cv*_ *=* population cross-validity coefficient*; Ȓ*^*2*^_*cv*_ *=* population squared cross-validity coefficient.

*p < .05.

**p < .01.

As can be seen, out of the three independent variables considered, conscientiousness appears as the strongest predictor of CAB with a relative predictive weight of β = -.64 (*p* < .01). The second-best predictor was agreeableness, with a beta value of β = -.45 (*p* < .01), followed by emotional stability with a result of β = .16 (*p* < .05). Altogether, conscientiousness, agreeableness, and emotional stability contribute to the explanation of 51% of CAB variance (*R*^2^ = .51, *R* = .71, *p* < .01). Consistent with the correlational results, conscientiousness and agreeableness have a negative impact on CAB, while emotional stability exerts a direct effect. As seen in Table 5 and in Fig 1, we also report the cross-validation coefficient (*Ȓ*_cv_) and the square cross-validation coefficient (*Ȓ*^2^_cv_). It is known that *R* and *R*^2^ can be biased due to capitalization on chance happening in multiple regression analyses. One of the consequences of such a phenomenon is that, if regression coefficients obtained in a given sample are applied to a different sample, the size of *R*^2^ will be lower than the estimate made in the first case. Furthermore, this effect is greater as the number of predictors included in the model increases. For this reason, the most accurate estimators to consider are the population cross-validity coefficient (*Ȓ*_cv_) and the population squared cross-validity coefficient (*Ȓ*^2^_cv_) [47,48]. The least biased formula for their calculation is the one developed by Browne [49]:

$${\hat{R}}_{Cv}^{2}=\frac{(N-k-3){\hat{R}}^{4}+{\hat{R}}^{2}}{(N-2k-2){\hat{R}}^{2}+k}$$ (1)

In the formula proposed, *N* is the number of observations, *k* is the number of predictor variables, *Ȓ*^2^ is obtained by using Formula 2, developed by Wherry [50] and *Ȓ*^4^ is obtained by applying Formula 3 derived by Lautenschlager [51].

$${\hat{R}}^{2}=1-(\frac{N-1}{N-k-1})(1-R^{2})$$ (2)

$${\hat{R}}^{4}=({{\hat{R}}^{2})}^{2}=\frac{2k{\left(1-{\hat{R}}^{2}\right)}^{2}}{\left(N-1)(N-k+1\right)}$$ (3)

The obtained coefficients were *Ȓ*_cv_ = .70 (*p* < 0.01) and *Ȓ*^2^_cv_ = .48.

## Discussion

This study has contributed to expand the knowledge on CAB in a context where primary research is severely lacking: the high school level of a country outside of the United States and Canada. The main objectives of this research were: (1) to examine the level of prevalence of overall CAB as well as its facets in this context; (2) to study the relationship of the Big Five model of personality and GMA with CAB and its facets; and (3) to develop an explicative model that summarizes the predictive effects of these individual differences on CAB.

Regarding the first goal, the results demonstrate that Spanish high school students often engage in counterproductive academic behaviors. The descriptive analyses of the CAB measure showed very high percentages of occurrence, indicating that most of the surveyed students have engaged in these negative practices during high school. One of the most worrisome aspects of the results is the fact that almost 83% and 82% of the sample acknowledges to having engaged in cheating and low effort behaviors at some point. Out of these rates, almost 17% and 21% of the sample confess to behaving in such ways always or almost always. These rates do not seem to differ much from empirical evidence gathered in the American context (described in the introduction section), nor are they very different from the occurrence rates found at other educational levels across the globe. For instance, the study by Teixeira and Rocha [52] reported on the percentage of college students engaging on deviant behaviors during examinations in different countries. Their findings yielded percentages as high as 83% in Brazil (*N* = 100), 71.6% in Austria (*N* = 519), 79.6% in Turkey (*N* = 528), or 84.6% in Slovenia (*N* = 321). Trost [53] found that 81% of 322 university students in Sweden had lied about a significant matter to get special treatment in the correction of their exams. Cuadrado, Salgado, and Moscoso [54] also report very similar results using a sample of 379 Spanish college students. The percentages of the students engaging in CAB at least once in college were 76.5%, 23.5%, 77.9%, 43.5%, and 76.2% for cheating, misuse of resources, absenteeism, breach of rules, and low effort behaviors, respectively.

In essence, the descriptive analyses indicated once again that CAB is a common phenomenon among high school students, with similar or even higher rates than those published in other countries and educational stages.

The next set of findings concerns the CAB correlates. Consistent with previous results, conscientiousness was the personality dimension most strongly linked to overall CAB and, especially, to three out of the five CAB facets (cheating, absenteeism, and low effort). Agreeableness was also a valid predictor of overall CAB and the best predictor of misuse of resources and breach of rules. Both dimensions appeared to be inversely linked to academic counterproductivity and yielded true effect sizes higher in magnitude than those reported in previous meta-analysis on this topic [see 17, 18]. For instance, there were .31 and .13 units of correlation of difference for conscientiousness and agreeableness, respectively, between the results found in the current research and the effect sizes published by Cuadrado et al. [17] using samples of high school students. This further backs up the hypothesis that even in high school, the more a student scores in conscientiousness and agreeableness, the less likely they are to engage in CAB.

In line with the findings by Cuadrado et al. [17] and supporting the research hypothesis, extraversion appeared as a direct and valid predictor of cheating behaviors. As it happened with conscientiousness and agreeableness, the true validity found in the current research was considerably larger (ρ = .19 vs. ρ = .43).

There were unexpected results referring to emotional stability. Neither meta-analyses by Giluk and Postlethwaite [18] nor by Credé et al. [16] found a link between this dimension and negative academic behaviors. In the meta-analysis of Cuadrado et al. [17] emotional stability appeared as a valid predictor of overall CAB at the high school level, however, the magnitude of the effect size was very low (ρ = .06). In the current research it seems that the most emotionally stable individuals are more prone to commit CAB than their unstable counterparts, especially regarding low effort behaviors, misuse of resources, and absenteeism. A possible explanation supporting this positive relationship might be the fact that emotional stable individuals tend to score high in traits such as tranquility, calmness, or imperturbability [20–23]. A higher score in these characteristics could make these individuals less prone to show a sense of urgency, to be more carefree, and hence, to have less qualms about not attending classes, not completing their classwork on time, not striving academically, or using academic supplies and equipment in an improper manner.

Regarding GMA, the results did not emerge as expected. GMA appeared as a weak predictor of overall CAB and its facets. In no case the results were significant. Additionally, the directionality was not as expected; except for the cheating facet, the validity coefficients were positive. These results are different to previous findings by Cuadrado et al. [17], Paulhus and Dubois [19], and Credé et al. [16]. Some possible explanations of these differences could be the sampling error effect or the existence of moderating variables that might be affecting the results. Consequently, more primary research on this relationship is needed.

The last goal of this research was to create an explicative model of CAB by incorporating the most powerful predictors found at the correlational level. The estimated model showed that the variables most highly linked to CAB (conscientiousness, agreeableness, and emotional stability) explain more than half of the CAB variance. Conscientiousness was the variable with the highest predictive weight, which is also consistent with previous findings [see 54].

### Suggestions for practitioners and future research

The empirical findings of the current research are important for applied purposes in the context of secondary education. First, academic administrators, faculty, and parents must be made aware of the fact that CAB is not an isolated problem affecting only a few specific academic institutions at certain academic levels in +a limited number of countries. Although research has been mostly performed in the higher education system of North America, empirical evidence indicates that it is a widespread phenomenon across the world that can be found in the lowest to the highest levels of education. The present study showed that levels of occurrence among the students of a Spanish high school are very high. For this reason, applied measures must be designed and taken into practice. Some of these deterrent measures are related to the variables examined in this study. It was shown that emotional stability, agreeableness, and especially conscientiousness predict CAB and its facets. The use of personality measures in secondary education cannot be conceived in the same way as in higher education or occupational contexts, where these instruments can be used to make high-stakes decisions (e.g., to determine access to a masters course, to a PhD program, or to an occupational position). However, knowing the personality profiles of the students, especially in small-sized classrooms, may be of some utility in high schools. The use of personality instruments could help identify those students with certain personality characteristics that, potentially, make them more likely to engage in CAB and, consequently, may need more personalized attention in the performance of certain academic activities like tests or examinations. This would reduce their chances of engaging in prohibited conducts and help increase the fairness of assessments by preventing dishonest students from getting a higher grade than they deserve.

Besides the use of personality measures as a preventive initiative, it has become more necessary than ever to promote additional integrity measures in high schools. As Bertram-Gallant and Drinan [55] state, systematic interventions performed by administrators, faculty, and students are needed to establish a climate of academic integrity. All the involved actors, especially students, must be aware of behaviors that qualify as CAB, the consequences of engaging in CAB, and the benefits of behaving in an honest manner. These actions may potentially reduce the prevalence of CAB.

The next suggestion refers to the response format of the personality measures. In the current study, a single-stimulus instrument was used to assess the Big Five dimensions of personality. This type of measures is widely used in the W/O psychology and the educational psychology field. However, they show substantial correlation with social desirability and impression management in students [56]. It is known that forced-choice inventories, especially quasi-ipsative tests, are a preferable option when it comes to control faking or social desirability [57–61]. Furthermore, quasi-ipsative personality inventories have shown a similar or a better predictive validity than personality tests with other formats in the prediction of important criteria in both occupational and educational contexts [62–64]. In the study of CAB, only Cuadrado et al. [50] have analyzed this question using a quasi-ipsative questionnaire for higher education students. Hence, it is necessary to examine whether predictive validity of the Big Five personality model is similar or higher at the high school level by using quasi-ipsative personality measures.

In regard to the social desirability concern, meta-analytical evidence indicates that students scoring higher in this variable also tend to underreport their engagement in cheating behaviors [41]. Despite the fact that percentages of engagement in CAB found in the current research were very high, results could be even higher if they were controlled for a measure of social desirability. Researchers should address this question at the high school level.

We also suggest making further efforts in the study of the intelligence-CAB relationship. Given that neither the magnitude nor the directionality of the results were the expected, we recommend researchers to study more in depth the link between these variables in the Spanish secondary education context.

It is also recommended to expand the study of CAB to other practices that have not been contemplated in the current research. Although we have examined a wide range of CAB behaviors, there are some other facets that need to be further studied. One example is plagiarism of written projects defined as *“submitting another person’s work as an original work or a project done by oneself but previously submitted in the past*, *as well as any other behavior that consists of the dishonest alteration of others’ work”* [17]. Levels of occurrence of such behavior are believed to have increased in recent years due to technological advances and the expanded use of the Internet in multiple phases of the students’ academic life. Thus, it would be interesting to replicate the current study and analyze plagiarism behaviors.

Additionally, we suggest testing some possible moderating variables that could have affected our results. For instance, the high schools that participated in this research were without exception public institutions. It could be interesting to replicate this research in private schools. Other contextual variables as well as individual differences other than personality and intelligence should be also addressed in future research.

### Limitations of the study

It is important to consider the limitations of this study. First, the time restrictions in the data collection made it impossible to administer all the instruments to a part of the sample. As a direct consequence, the sample size for the personality measure was smaller than the sample size obtained for the GMA and CAB measures. It is known that small samples increase sampling error, causing a random variation of the observed validity from the true validity [42]. Also, because the sampling error is unsystematic, it cannot be corrected in a single correlation and there is no possibility to control its effects unless the validity coefficients are integrated in a meta-analysis, hindering the replicability of the results as well.

A second potential limitation is that the questionnaires were not anonymous. Although the rates of engagement in counterproductivity were very high, it is possible that anonymity would yield even higher prevalence levels, especially in those dimensions where rates were lower (e.g., misuse of resources).

## Conclusion

The current research does a contribution on the study of CAB and its facets in a context where research is not very prominent: the secondary education outside of the United States and Canada. The findings indicate that the prevalence of these practices is as alarming as in other countries and educational levels and are a wake-up call for academic administrators to seriously address this problem. Results also show that students’ personality characteristics account for CAB variance, especially emotional stability, agreeableness, and conscientiousness, the latter being the most relevant both at the correlational level and in the regression analyses. We encourage future researchers on this topic to replicate our results using high school samples from other geographical areas.
